# Placental Barrier Breakdown Induced by *Trypanosoma cruzi*-Derived Exovesicles: A Role for MMP-2 and MMP-9 in Congenital Chagas Disease

**DOI:** 10.3390/ijms262412131

**Published:** 2025-12-17

**Authors:** Alejandro Fernández-Moya, Ana Liempi, Marioly Müller, Rocío Arregui, Antonio Osuna, Alberto Cornet-Gómez, Christian Castillo, Ulrike Kemmerling

**Affiliations:** 1 Núcleo Interdisciplinario de Biología y Genética, Instituto de Ciencias Biomédicas (ICBM), Facultad de Medicina, Universidad de Chile, Santiago 8380000, Chile; alefernandez@ug.uchile.cl (A.F.-M.); anitavet@gmail.com (A.L.); rocio.arregui@ug.uchile.cl (R.A.); 2 Instituto de Ciencias Naturales, Facultad de Medicina Veterinaria y Agronomía, Universidad de Las Américas, Santiago 9250000, Chile; 3 Departamento de Tecnología Médica, Facultad de Medicina, Universidad de Chile, Santiago 8380000, Chile; marioly@uchile.cl; 4 Departamento de Parasitología, Instituto de Biotecnología, Universidad de Granada, 18071 Granada, Spain; aosuna@ugr.es (A.O.); acornetgomez@ugr.es (A.C.-G.)

**Keywords:** *Trypanosoma cruzi*, congenital transmission, extracellular vesicles, placenta, matrix metalloproteinases 2 and 9

## Abstract

*Trypanosoma cruzi*, the causative agent of Chagas disease, can cross the placental barrier and be transmitted congenitally, yet the mechanisms underlying this process remain incompletely understood. Recent evidence suggests that *T. cruzi*-derived extracellular vesicles (TcEVs) may facilitate placental invasion by modulating host–pathogen interactions. In this study, we examined the effects of TcEVs on human placental explants (HPEs), focusing on their capacity to disrupt tissue architecture and modulate matrix metalloproteinases MMP-2 and MMP-9, enzymes critical for extracellular matrix remodeling. Term placental chorionic villi were cultured ex vivo and exposed to TcEVs, heat-inactivated TcEVs, infective trypomastigotes, or combinations thereof. TcEVs induced ultrastructural damage, including trophoblast detachment and basal lamina disorganization, which were exacerbated by co-incubation with parasites. Immunohistochemistry and Western blotting revealed significant upregulation of MMP-2 and MMP-9, while gelatin zymography confirmed increased enzymatic activity. Our findings demonstrate that TcEVs independently and synergistically with *T. cruzi* compromise placental integrity by enhancing MMP expression and activity, thereby priming the placental microenvironment for parasite invasion. Targeting TcEVs signaling or MMP activation may represent a novel strategy to prevent congenital transmission of *T. cruzi*.

## 1. Introduction

Chagas disease (CD), caused by the protozoan *Trypanosoma cruzi* (*T. cruzi*), remains a major neglected tropical disease with an estimated 6–7 million infections globally. While historically endemic to Latin America, CD now presents a growing burden in non-endemic regions due to migration and globalization [1,2,3,4,5,6]. In this context, congenital transmission, where the parasite breaches the placental barrier, has emerged as the primary non-vectorial route of infection, particularly among pregnant women of Latin American origin [2,5,7,8,9].

The placenta constitutes a sophisticated, multilayered defense system against pathogens. Its architectural core resides in the chorionic villi, where a bilayer of trophoblasts (cytotrophoblast and syncytiotrophoblast), a continuous basal lamina, and a villous stroma (VS) rich in extracellular matrix (ECM) collaboratively shield the fetus [1,3,10,11,12,13,14,15]. Successful congenital transmission requires *T. cruzi* to disrupt this barrier, a process mediated by parasite-derived virulence factors delivered via extracellular vesicles (TcEVs) [1,3,11,16,17].

These lipid bilayer nanoparticles, secreted by trypomastigotes, carry molecular cargo including trans-sialidases (TSs), mucin-associated surface proteins (MASPs), and cruzipain (Cz) that subvert host cell functions to promote adhesion, invasion, and immune evasion [16,18,19,20,21]. Critically, our recent evidence demonstrates that TcEVs alone induce structural damage and apoptosis in human placental explants (HPEs) [3].

A key vulnerability exploited during placental invasion is dysregulation of ECM homeostasis. The ECM not only provides structural integrity but also modulates trophoblast differentiation and immune responses [22,23,24]. Matrix metalloproteinases (MMPs), particularly the gelatinases MMP-2 and MMP-9, are pivotal regulators of ECM remodeling during pregnancy [22,25,26]. Physiologically, their activity is tightly controlled; however, pathological overexpression correlates with pregnancy complications, including preterm rupture of membranes, preeclampsia, and microbial infections [23,25,27,28,29]. Evidence from ex vivo models indicates that *T. cruzi* increase the expression and enzymatic activity of MMP-2 and MMP-9 in HPEs, contributing to basal lamina degradation and villous stroma disorganization [25].

Despite these insights, the precise molecular mechanisms by which TcEVs interact with placental tissues and modulate MMP activity remain incompletely understood. This study aims to investigate the role of TcEVs in modulating the expression and activity of MMP-2 and MMP-9, as well as the associated ultrastructural changes, during the ex vivo infection of HPEs. Understanding these mechanisms may help identify novel therapeutic targets to prevent congenital transmission of *T. cruzi* and improve outcomes for affected newborns.

## 2. Results

### 2.1. TcEVs Exacerbate Ultrastructural Damage to the Placental Barrier

**Figure 1:**
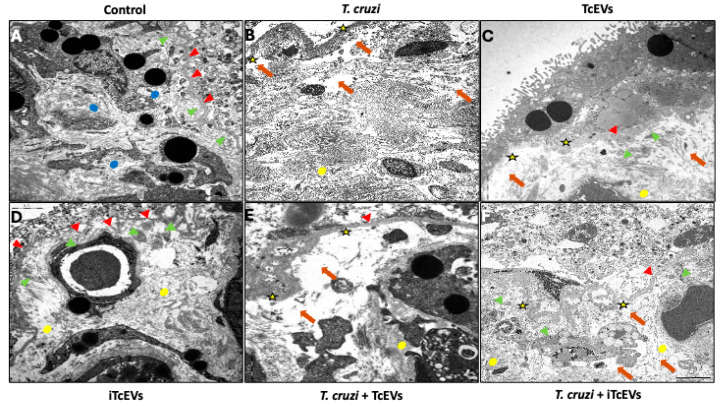
TcEVs disrupt the ultrastructure of the placental barrier in HPEs: HPEs were incubated for 24 h in the presence or absence of TcEVs (active or heat-inactivated) and/or infective trypomastigotes (10^5^ parasites/mL). Transmission electron microscopy revealed that control explants (**A**) and those treated with heat-inactivated TcEVs (iTcEVs) (**D**) maintained normal villous architecture, including a continuous basal lamina (red arrowheads), preserved anchoring between basal and reticular lamina (green arrowheads), and organized collagen fibers in the villous stroma (blue dots). Explants incubated with *T. cruzi* (**B**), *T. cruzi* and iTcEVs (**F**) or TcEVs (**C**) displayed basal lamina disorganization (yellow stars) and stromal degradation (red arrows) including collagen I disorganization (yellow dots), with more severe alterations observed in co-incubated explants (**E**). Scale bar: 2 μm.

HPEs exposed to *T. cruzi* trypomastigotes (10^5^ parasites/mL), TcEVs, or their combination for 24 h exhibited profound ultrastructural alterations visualized by electron microscopy (Figure 1). Control HPEs (Figure 1A) and those incubated with heat-inactivated TcEVs (iTcEVs; Figure 1D) maintained architectural integrity, characterized by a continuous basal lamina of uniform thickness (red arrowheads), preserved anchoring complexes between the lamina densa and reticular lamina (green arrowheads), and well-organized collagen fibrils in the villous stroma (blue dots). In stark contrast, *T. cruzi*-exposed explants (Figure 1B) displayed near-total trophoblast detachment, accompanied by basal lamina thickening with spiculated contours (yellow stars) and stromal voids (orange arrows) indicative of three-dimensional matrix collapse that included collagen I disorganization (yellow dots). Explants treated solely with TcEVs (Figure 1C) showed intermediate pathology, featuring focal trophoblast detachment and basal lamina thinning without complete structural dissolution. Critically, co-incubation with *T. cruzi* and TcEVs (Figure 1E) synergistically amplified these defects, resulting in extensive trophoblast loss, basal lamina fragmentation, and pervasive stromal disintegration. These findings establish that TcEVs autonomously disrupt placental integrity and potentiate parasite-induced barrier damage.

### 2.2. TcEVs per se Increase MMP-2 and MMP-9 Expression in the Villous Stroma

To examine the role of TcEVs in modulating ECM remodeling within the placental microenvironment, we evaluated the expression of matrix metalloproteinases MMP-2 and MMP-9 in HPEs.

**Figure 2:**
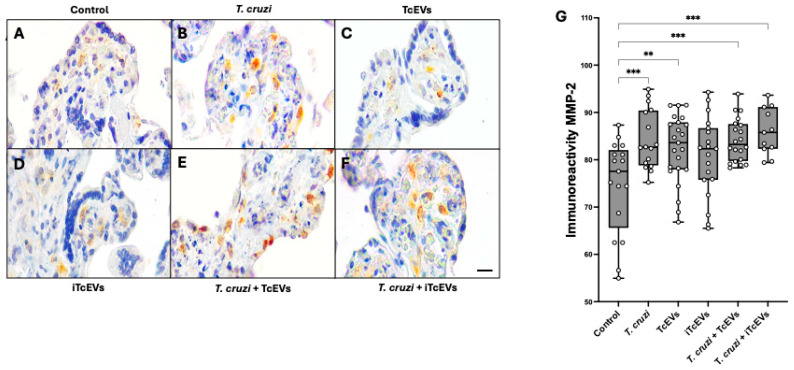
TcEVs increase MMP-2 expression in HPEs: Paraffin-embedded HPEs were subjected to immunohistochemical staining using anti-MMP-2 antibodies after 24 h of incubation with TcEVs, iTcEVs, *T. cruzi*, or their combinations. Panels **A**–**F** show representative images: Control (**A**), *T. cruzi* (**B**), TcEVs (**C**), iTcEVs (**D**), *T. cruzi* + TcEVs (**E**), and *T. cruzi* + iTcEVs (**F**). Quantification of MMP-2 immunoreactivity (**G**) was conducted by image analysis of DAB staining, expressed as the reciprocal of RGB intensity in a 0–255 scale. A significant increase in MMP-2 expression was detected in conditions including *T. cruzi* and/or TcEVs. N = 3 placentas in triplicate. Scale bar: 25 μm. * *p* < 0.05, ** *p* < 0.01, *** *p* < 0.001, **** *p* < 0.0001 by one-way ANOVA followed by Dunnett’s test.

**Figure 3:**
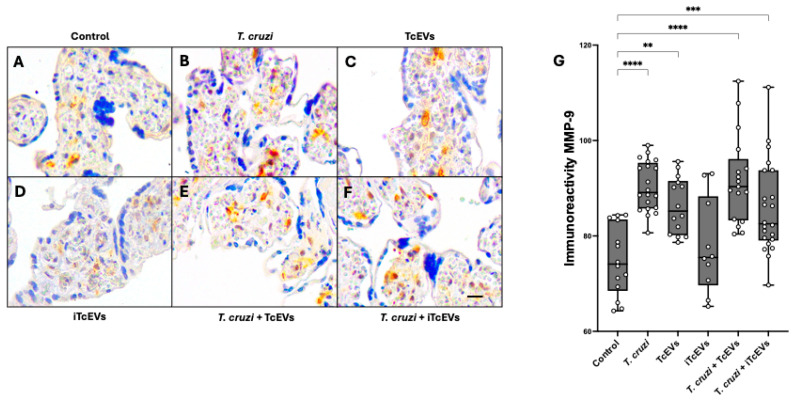
TcEVs increase MMP-9 expression in HPEs: Paraffin-embedded HPEs were subjected to immunohistochemical staining using anti-MMP-9 antibodies after 24 h of incubation with TcEVs, iTcEVs, *T. cruzi*, or their combinations. Panels **A**–**F** show representative images: Control (**A**), *T. cruzi* (**B**), TcEVs (**C**), iTcEVs (**D**), *T. cruzi* + TcEVs (**E**), and *T. cruzi* + iTcEVs (**F**). Quantification of MMP-9 immunoreactivity (**G**) was conducted by image analysis of DAB staining, expressed as the reciprocal of RGB intensity in a 0–255 scale. A significant increase in MMP-9 expression was detected in conditions including *T. cruzi* and/or TcEVs. N = 3 placentas in triplicate. Scale bar: 25 μm. * *p* < 0.05, ** *p* < 0.01, *** *p* < 0.001, **** *p* < 0.0001 by one-way ANOVA followed by Dunnett’s test.

Basal immunoreactivity for both MMP-2 (Figure 2A) and MMP-9 (Figure 3A) was observed across all conditions, as expected due to their physiological expression in mesenchymal cells such as fibroblasts within the VS. In control HPEs (A) and those incubated with heat-inactivated TcEVs (iTcEVs) (D), staining for both MMPs appeared faint and localized, indicating low basal expression. In contrast, samples exposed to *T. cruzi* or TcEVs alone showed a significant increase in immunoreactivity for both MMP-2 and MMP-9 (B,C), with more intense and diffuse immunolabeling localized mainly in the villous stroma. The strongest immunoreactivity was observed in the co-incubation condition (*T. cruzi* + TcEVs), where both MMP-2 and MMP-9 signals appeared markedly increased (E).

Quantitative image analysis of staining intensity supported these qualitative findings. For MMP-2 (Figure 2G), explants incubated with *T. cruzi* showed a significant increase of 9.77 ± 2.52 units (*p* = 0.0009) compared to controls. TcEVs alone induced an increase of 7.80 ± 2.31 (*p* = 0.0049), while the *T. cruzi* + TcEVs group exhibited increases of 9.64 ± 2.39 (*p* = 0.0005). A similar pattern was observed for MMP-9 (Figure 3G). The *T. cruzi* group showed an increase of 15.53 ± 2.81 (*p* < 0.0001) compared to controls, while TcEVs alone led to an increase of 11.48 ± 3.05 (*p* = 0.0013). Co-incubation with *T. cruzi* and TcEVs further elevated MMP-9 expression to 16.90 ± 2.91 (*p* < 0.0001). The iTcEVs also induced a moderate but not significant increase in MMP-2 (11.44 ± 2.80 (*p* = 0.0004)), and of MMP-9 (10.91 ± 2.76 (*p* = 0.0007)), respectively.

**Figure 4:**
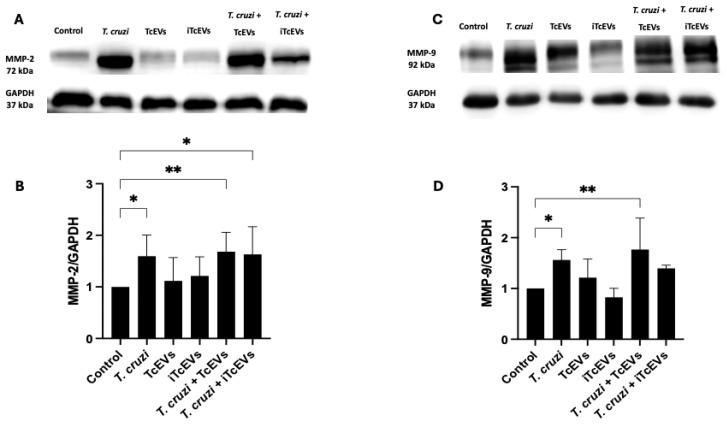
TcEVs increase MMP-2 and MMP-9 protein expression in HPEs: Western blot analysis was performed on lysates of HPEs incubated under the same experimental conditions. Representative blots showing (**A**) MMP-2, (**C**) MMP-9, and GAPDH expression. Densitometric analysis (**B**,**D**) revealed increased expression of both MMP-2 and MMP-9, especially in conditions including *T. cruzi* and its combination with TcEVs. TcEVs alone also showed a trend toward increased MMPs expression, though not statistically significant. Data represent mean ± SD. * *p* < 0.05, ** *p* < 0.01 by one-way ANOVA with Dunnett’s post-test.

To further quantify the expression levels of MMP-2 and MMP-9, Western blot analysis was performed to assess MMP protein levels following exposure to *T. cruzi*, TcEVs, or both (Figure 4). Compared to control explants, a significant increase in MMP-2 (Figure 4A,B) and MMP-9 (Figure 4C,D) expression was observed in the group incubated with *T. cruzi*, showing a densitometric fold-change of 59.5 ± 16.7% (*p* = 0.0194) and 56.1 ± 10.8% (*p* = 0.0405), respectively. Similarly, co-incubation with *T. cruzi* and TcEVs increased MMP-2 (68 ± 15.23% (*p* = 0.0059)) and MMP-9 (76.7 ± 28.8% (*p* = 0.0085)). However, explants treated with TcEVs alone exhibited only a mild increase in MMP-2 (12.1 ± 16.6%) and MMP-9 (21.3 ± 18.28) expression, which was not statistically significant when compared to uninfected controls. These findings suggest that while TcEVs alone may exert subtle regulatory effects on MMP levels, their biological impact on protein expression becomes more evident in the presence of the parasite, highlighting a potential cooperative mechanism in ECM remodeling.

### 2.3. TcEVs Modulate Gelatinolytic Activity of Endogenous MMPs in HPEs

**Figure 5:**
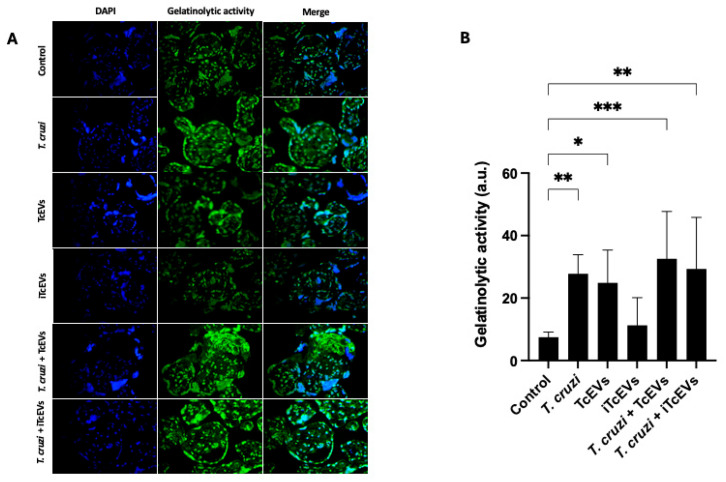
TcEVs enhance gelatinolytic activity of MMPs in HPEs: Gelatin in situ zymography was used to detect the enzymatic activity of MMPs in HPEs exposed to TcEVs, iTcEVs, *T. cruzi*, or their combinations for 24 h. Representative fluorescence images (**A**) demonstrate increased green fluorescence (gelatinolytic activity) in explants incubated with *T. cruzi* and/or TcEVs. Quantification (**B**) confirmed significant increases in enzymatic activity compared to the control and iTcEVs groups. Doxycycline (1 μM) was used as a negative control for enzymatic activity. N = 3 placentas in triplicate. * *p* < 0.05, ** *p* < 0.01, *** *p* < 0.001 by one-way ANOVA with Dunnett’s and Tukey’s post-tests.

To functionally evaluate the enzymatic activity of MMPs in HPEs after exposure to *T. cruzi* and TcEVs, a gelatinolytic activity assay was performed (Figure 5).

Control HPEs and those incubated with iTcEVs displayed low basal gelatinolytic activity, as reflected by faint fluorescence intensity. In contrast, explants incubated with *T. cruzi* or TcEVs exhibited a significant increase in gelatinase activity, with higher fluorescence localized to both the trophoblastic layer and villous stroma. This increase was even more pronounced in explants co-incubated with *T. cruzi* and TcEVs, suggesting an additive effect in the activation of MMPs (Figure 5A).

Quantitative analysis of the fluorescence signal confirmed these observations (Figure 5B). Samples incubated with *T. cruzi* (372.17% ± 60.89 (*p* = 0.0036)), in combination with TcEVs (436.55% ± 151.70 (*p* = 0.0036)) or TcEVs alone (333.19% ± 105.00 (*p* = 0.0396)), demonstrated statistically significant increases in MMP activity when compared to controls, supporting the biological relevance of vesicle-mediated modulation of ECM-degrading enzymes.

## 3. Discussion

Our study demonstrates that TcEVs, derived from *T. cruzi*, significantly compromise the structural integrity of the human placental barrier by upregulating key matrix metalloproteinases, MMP-2 and MMP-9. These enzymes, involved in ECM remodeling, were elevated at the protein level and showed increased gelatinolytic activity in ex vivo infected HPEs exposed to *T. cruzi*, TcEVs, or both, highlighting TcEVs as active agents of placental damage and potential facilitators of parasite transmission.

Anatomical barriers, including the skin, mucosal surfaces, and notably the placental barrier, form the first line of defense in innate immunity, structuring a multilayered shield against infection [11,14,17,30,31,32,33]. Here, our ultrastructural analysis revealed that exposure to TcEVs leads to partial detachment of trophoblasts, thinning of the basal lamina, and disorganization of collagen fibrils within the villous stroma (Figure 1). When co-incubated with the parasite, these effects are markedly amplified, resulting in widespread stromal collapse and breakdown of the barrier, supporting previous findings showing that TcEVs can induce apoptosis, cytoskeletal disarray, and syncytiotrophoblast destruction and detachment in placental tissue as well as collagen I disorganization, even in the absence of the parasites [3]. Such structural alterations compromise the primary anatomical and immunological defenses of the placenta, increasing its permeability to pathogens [1,11,14,17,31,34]. The ability of TcEVs to damage placental architecture likely arises from their cargo of virulence factors, including TSs, MASPs, and Cz, which are capable of modulating host cell signaling, promoting ECM degradation, and evading immune detection [16,18,35,36,37]. Cz, the main parasite-derived proteases have been associated with tissue destruction and invasion [38,39]. Moreover, Cz and other TcEVs cargo molecules modulate host cell signaling [38,40,41]. On the other hand, TcEVs might modulate MMPs indirectly. MMPs can be induced and activated in a pro-inflammatory environment [42,43] and, in turn, TcEVs induce severe inflammation in different experimental models [44,45,46,47,48].

Immunohistochemistry analyses revealed that TcEVs alone significantly increased MMP-2 and MMP-9 expression in HPEs, and the presence of the parasites further enhanced this effect. This finding aligns with prior studies demonstrating upregulation of these gelatinases during *T. cruzi* infection [25]. On the other hand, MMP-2 and MMP-9 play essential roles in placental ECM turnover during normal pregnancy; however, their dysregulation has been implicated in pathological processes such as preterm labor [26,28,49] and vertical transmission of viral infections [27,50,51]. Thus, given their capacity to degrade type IV collagen and laminin in the basal lamina, increased MMP activity in TcEV-treated explants likely contributes to trophoblast detachment and stroma disorganization. These results mirror placental damage observed in congenital infections caused by *Cytomegalovirus* [52], *T. gondii* [53] and Zika virus [27].

Beyond the upregulation in protein expression, gelatin zymography confirmed that TcEVs also activate MMPs enzymatically. Notably, heat-inactivated TcEVs lost this ability, suggesting that heat-labile parasite-derived proteins or enzymes are involved in the activation process. This observation aligns with prior work demonstrating that TcEVs modulate host physiology through their proteolytic content [54,55,56,57,58].

It is also plausible that TcEVs carry miRNAs or signaling lipids that indirectly activate latent MMPs via host signaling pathways. Such vesicle-mediated crosstalk is increasingly recognized in other parasitic diseases and may represent a conserved strategy for host modulation [38,59,60,61,62].

Our findings support a model where TcEVs act as paracrine effectors that precondition the placental microenvironment for successful parasite invasion. By degrading ECM components and weakening trophoblast attachment, TcEVs likely lower the physical and immunological threshold for parasite traversal across the maternal-fetal interface, explaining how congenital transmission can occur even in women with low parasitemia and no overt placental inflammation [2]. Such a mechanism is reminiscent of the “pathogen priming” strategy described in other congenital infections, where secreted or EV-associated virulence factors initiate subtle but progressive tissue remodeling before overt infection [27,44,56,58,63].

**Figure 6:**
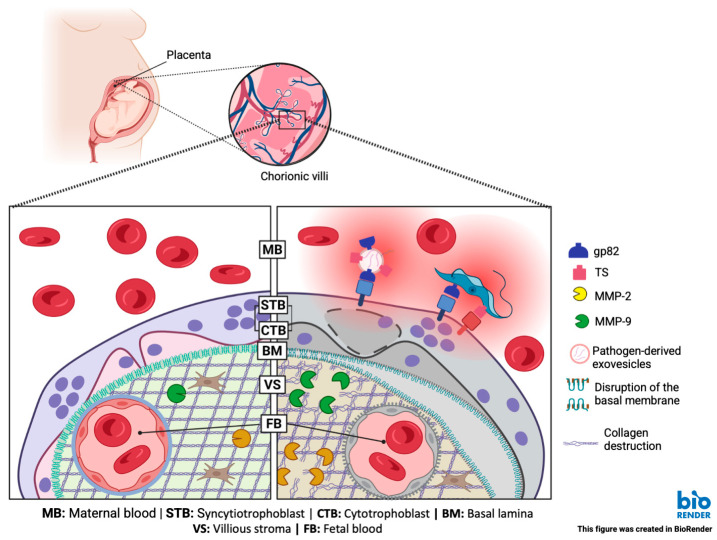
Proposed model of TcEV-induced disruption of the placental barrier: Schematic illustration depicting the pathogenic mechanisms by which *Trypanosoma cruzi*-derived exovesicles (TcEVs) compromise placental integrity. Exovesicles released by trypomastigotes carry virulence factors such as gp82 and trans-sialidases (TSs), which interact with the syncytiotrophoblast (STB) and cytotrophoblasts (CTBs) of the chorionic villi. These interactions stimulate the expression and activation of matrix metalloproteinases MMP-2 and MMP-9, leading to degradation of the basal lamina (BM) and extracellular matrix components, such as collagen, within the villous stroma (VS). This ECM remodeling promotes trophoblast detachment and enhances the risk of vertical parasite transmission across the maternal-fetal interface. MB: maternal blood; FB: fetal blood. Figure created using BioRender (https://www.biorender.com/, last accessed on 10 December 2025).

To visually summarize our findings and propose a mechanistic model, we generated a schematic representation of TcEV-mediated placental barrier disruption (Figure 6). This model illustrates the sequence of molecular and structural events triggered by TcEVs upon interaction with the chorionic villi. Key components of the TcEV cargo could modulate host cell signaling and adhesion, facilitating the upregulation of matrix metalloproteinases MMP-2 and MMP-9. These enzymes degrade critical extracellular matrix components leading to the destruction and detachment of the trophoblast layer and disorganization of the VS, weakening the placental barrier.

A key strength of this study is the use of ex vivo infection of HPEs, which preserves the native ECM and trophoblast architecture, often lost in cell-line models [64].

However, our study has limitations. The ex vivo model lacks maternal immune components, which may interact with TcEVs or MMPs in vivo. We did not assess downstream immune signaling or TIMP (tissue inhibitor of metalloproteinases) activity, which could counterbalance the effects of MMP. Furthermore, while protein levels and activity of MMPs were quantified, the signaling pathways mediating their upregulation remain to be elucidated. Other limitation is the fact that we do not know the exact concentration of TcEVs that could be circulating in a pregnant woman with Chagas disease. Previous studies have evidenced that in patients with Chagasic cardiomyopathy, the total amount of exovesicles is lower than in healthy patients (2 × 10^8^ vs. 4 × 10^8^) [65]. However, the percentage of these exovesicles that corresponds to TcEVs is unknown. In addition, the concentration of circulating TcEVs also depends on the patient’s parasitemia, which varies between the acute and chronic phases [66]. The quantity of parasites used in this study considers a parasitemia of 0.1–1 parasite/mL [67] and the increased cardiac output in pregnant women [68].

Future studies should explore the specific molecular components of TcEVs responsible for MMP activation using proteomic or RNA sequencing approaches. The use of MMP inhibitors or genetic silencing tools in *ex vivo* or *in vivo* models would clarify whether MMPs are necessary for TcEV-induced damage. It would also be valuable to investigate whether maternal inflammation, coinfections, or hormonal changes modulate the placental response to TcEVs, influencing transmission risk.

Therapeutically, targeting TcEV biogenesis, cargo sorting, or host uptake pathways may offer novel strategies to reduce congenital transmission. Similarly, circulating TcEVs or their components could serve as biomarkers of fetal risk in pregnant women with *T. cruzi* infection.

## 4. Materials and Methods

### 4.1. Parasite Culture and Harvesting

Infective trypomastigote forms of *T. cruzi* were obtained by replicating part of their biological cycle in VERO cells (ATCC^®^ CCL-81, Manassas, MA, USA). VERO cells were grown in RPMI medium (Gibco, New York, NY, USA) supplemented with 5% fetal bovine serum (FBS) and antibiotics (penicillin-streptomycin). Semiconfluent VERO cells were incubated with a culture of Y-strain epimastigotes (a non-infective cellular form of the parasite) in the late stationary phase containing about 5% of infective trypomastigotes. Trypomastigotes invade fibroblasts and replicate intracellularly as amastigotes. After 72 h, amastigotes transform into trypomastigotes, which lyse the host cells. The parasites were recovered by low-speed centrifugation (500× *g*), resulting in trypomastigotes in the supernatant and amastigotes in the sediment [69]. The laboratory is certified as a A2 biosafety level by the “Unidad de Prevención de Riesgo” of the faculty of Medicine at the University of Chile.

### 4.2. EV Isolation and Treatments

Briefly, *T. cruzi* purified trypomastigotes were incubated for five hours at 37 °C in RPMI medium (Sigma Aldrich^®^, Burlington, MA, USA) buffered with 25 mM HEPES at 7.2 and supplemented with 10% exosome-free IFBS. Afterward, parasites were removed by centrifugation at 3500× *g* for 15 min; the supernatant was collected and centrifuged at 17,000× *g* for 30 min at 4 °C to eliminate the apoptotic bodies and ectosomes and then filtered through a 0.22 μm pore filter (Sartorius, Germany) and ultracentrifuged at 100,000× *g* for 16 h to obtain the TcEVs. The resulting pellet was washed three times in PBS by ultracentrifugation and resuspended in 100 μL of PBS. To inactivate parasite virulence factors, TcEVs were incubated in a water bath at 80 °C for 30 min and then washed twice in PBS by ultracentrifugation at 100,000× *g* for 1 h [54,55]. The size and concentration of TcEVs samples were determined by measuring Brownian motion as a function of particle size using a NanoSight NS300 (Malvern Instruments, Great Malvern, UK), a system equipped with an sCMOS camera and a 488 nm blue laser beam, as described previously [70]. Inactivation of the TcEVs was assayed by protease enzymatic activity. Briefly, TcEVs were resuspended in a solution containing 1 mL of N-α-benzoyl-DL-arginine (BApNA) N-α-benzoyl-DL-arginineHCl (pH 7.4), and then 0.2 mM Dithiothreitol was added. The samples were incubated at 37 °C for 30–60 min of reaction, and finally, the absorbance of each solution was measured at 405 nm [3]. The iTcEVs did not show protease activity. TvEVs morphology and size was analyzed by Transmission electron microscopy (TEM) (ZEISS EM 902, Jena, Germany) and by nanoparticle tracking analysis (NTA) (NanoSight Ltd., Amesbury, UK), respectively (Supplementary Figure S1).

### 4.3. HPE Culture and Parasite Infection

Human-term placentas were obtained from uncomplicated pregnancies from cesarean deliveries. The study was conducted in accordance with the Declaration of Helsinki and approved by the Institutional Ethics Committee of Facultad de Medicina, Universidad de Chile (Number 042-2022, 25 May 2002). Each patient provided informed consent for the experimental use of the placenta, as stipulated by the Code of Ethics of the Faculty of Medicine at the University of Chile. The exclusion criteria for the patients were the following: major fetal abnormalities, placental tumor, intrauterine infection, obstetric pathology, or any other maternal disease. The organs were collected in a cold, sterile, saline-buffered solution (PBS) and processed within 30 min after delivery. The maternal and fetal surfaces were discarded, and villous tissue was obtained from the central part of the cotyledons. The isolated chorionic villi were washed with PBS to remove blood, dissected into approximately 0.5 cm^3^ fragments [53,71,72], and co-cultured for 24 h in the presence and absence of 0.2 µg/mL of TcEVs (equivalent to 0.284 × 10^8^ EVs/mL) or inactivated TcEVs (iTcEVs) and then in presence and absence of 10^5^ parasites/mL [3].

### 4.4. Transmission Electron Microcopy

HPEs were fixed for overnight in 3% glutaraldehyde in 0.1 M neutral phosphate buffer (pH 7.2) at 4 °C. Then, the HPEs were washed in Sodium phosphate buffer 0.1 M pH 7.2 at room temperature (×3 during 5 min). Samples were stained with 1% osmium tetroxide for one hour, washed (×3) in Sodium phosphate buffer 0.1 M pH 7.2, dehydrated in a graded ethanol proanalysis series (30%, 50%, 70%, 90%, 100% (×3); and finally, in pure acetone proanalysis (×3); 7 min each). Then, the HPEs were embedded in EPON epoxy resin-acetone 1:1 for 1 h, in EPON epoxy resin-acetone (3:1) for 4 h and, finally EPON epoxy resin 100% overnight at room temperature. After leaving the tissue overnight in EPON epoxy resin at room temperature, samples were incubated for 2 days at 70 °C. Finally, once the blocks were obtained, they were processed with an ultramicrotome Porter blum. Semithin sections (1 µm) were obtained stained with toluidine blue (1%) in sodium borate (4%), and observed under an light microscopy (Leica DM500, Weztlar, Germany)). The areas of interest were selected and trimmed to obtain ultra-thin sections (60 nm). Grids were dual-contrasted with uranyl acetate (2%) in water and lead citrate Reynolds solution (10 min each), and then analyzed and photographed on a Jeol 1010 Electron Microscope (Jeol Ltd., Tokio, Japan) at an accelerating voltage of 80–100 kV [73].

### 4.5. MMP Immunohistochemistry

The HPE were fixed in 4% paraformaldehyde (PFA) in 0.1 M phosphate buffer (pH 7.3) for 24 h, then dehydrated in alcohol, clarified in xylene, embedded in paraffin, and sectioned at 3 µm. Standard immunoperoxidase techniques were used to show MMP-2 (Cell Signaling^®^ (Danvers, MA, USA) 40994S, 1:200 *v*/*v*), and MMP-9 (Cell Signaling^®^ (Danvers, USA) 13667S, dilution 1:200 *v*/*v*). Briefly, the primary antibodies were applied individually to each section for one hour at 4 °C overnight. Immunostaining was performed using a horseradish peroxidase-labeled streptavidin-biotin kit (ImmPACT DAB Peroxidase Substrate #SK-4105; Vector Laboratories (Newark, CA, USA)) according to the manufacturer’s instructions, with diaminobenzidine as the chromogen. Sections were counterstained with Mayer’s hematoxylin (ScyTek, Logan, UT, USA) and mounted with Entellan (Merck, Kenilworth, NJ, USA). Immunohistochemical controls were performed by replacing the primary antibodies with phosphate-buffered saline [29,71,74]. All controls were negative. All sections were examined by light microscopy (Leica DM500 (Leica, Weztlar, Germany)), and images were captured with a Leica ICC50W camera (Leica, Weztlar, Germany). The chromogen intensity in immunohistochemistry and histochemistry was analyzed using reciprocal staining with Fiji-ImageJ2 (http://fiji.sc/Fiji, last accessed on 10 December 2025) software. At least ten sections from different areas of three different slides in each experimental condition were used. We counted five squares of approximately 4000 μm^2^ in each section [3,75].

### 4.6. MMP Detection by Western Blotting

Tissues were homogenized in a lysis buffer (Tris 10 mM, pH 8.0; SDS 1% *w*/*v*) and protease inhibitor cocktail (Complete Mini (Roche^®^, Basilea, Switzerland)) at 4 °C using a Potter Elvejem homogenizer, and the homogenate was centrifuged at 15,000× *g* for 20 min to remove debris. Protein concentration was determined using the Bradford assay, with bovine serum albumin as the protein standard. 30 mg of protein were separated in a 10% sodium dodecyl sulfate polyacrylamide gel, blotted onto a nitrocellulose membrane, and probed with monoclonal antibodies against MMP-2 (Cell Signaling^®^ (Danvers, MA, USA) 40994S; 1:1000, *v*/*v*) or MMP-9 (Cell Signaling^®^ (Danvers, USA) 13667S, 1:1000 *v*/*v*). To correct for loading, membranes were stripped and reprobed with an anti-rabbit GAPDH (Invitrogen MA5-15738 (Waltham, MA, USA), 1:5000 *v*/*v*) antibody. Immunoreactive proteins were detected using enhanced chemiluminescence reagents in a membrane scanner (Li-cor C-Digit, Lincoln, NE, USA) according to the manufacturer’s instructions (Amersham Biosciences Ltd., Amersham, UK). The films were scanned, and the NIH Image software program, version 1.6 (NIH, Bethesda, MD, USA), was used for densitometric analysis of the bands [25].

### 4.7. Gelatin In Situ Zymography

HPEs were fixed in anhydrous acetone (−20 °C) and processed for paraffin embedding (see above). The localization of the gelatolytic activity was determined using the Invitrogen DQ Gelatin kit according to the manufacturer’s instructions (Carlsbberg, New Canaan, CT, USA), and then counterstained with DAPI (4′,6-diamidino-2-phenylindole) [75]. Fluorescence was visualized using a Motic BA-310 epifluorescence microscope (Xiamen, China) equipped with a digital camera and analyzed using the Fiji-ImageJ software (http://fiji.sc/Fiji, last accessed on 10 December 2025). At least five sections from different areas of three different slides in each experimental condition were used.

### 4.8. Statistics

All experiments were triplicated in at least three placentas. Results are expressed as means ± SD. The significance of differences was evaluated using Student’s *t*-test for paired data or by ANOVA followed by Dunnett’s post hoc test.

## 5. Conclusions

This study reveals that TcEVs compromise placental integrity by inducing structural disruption and upregulating MMP-2 and MMP-9 expression and activity. These changes weaken the placental barrier and likely facilitate parasite translocation, contributing to congenital transmission.

## Data Availability

The original contributions presented in this study are included in the article/Supplementary Materials. Further inquiries can be directed to the corresponding author.

## Supplementary Materials

The following supporting information can be downloaded at: https://www.mdpi.com/article/10.3390/ijms262412131/s1.

## Abbreviations

The following abbreviations are used in this manuscript:

CD	Chagas disease
*T. cruzi*	*Trypanosoma cruzi*
VS	Villous stroma
ECM	Extracellular matrix
TcEVs	*T. cruzi*-derived extracellular vesicles
TS	Transialidase
MASPs	Mucin-associated Surface proteins
Cz	Cruzipain
HPEs	Human placental explants
MMP-2	Matrix metalloproteinase-2
MMP-9	Matrix metalloproteinase-9
iTcEVs	Heat-inactivated *T. cruzi*-derived extracellular vesicles
*T. gondii*	*Toxoplasma gondii*
FBS	Fetal bovine serum
IFBS	Inactivated fetal bovine serum
PBS	Phosphate-buffered saline
PFS	Paraformaldehyde
DAB	Diaminobenzidine
DAPI	4′,6-diamidino-2-phenylindole
DL-BAPNA	Nα-Benzoyl-DL-arginine 4-nitroanilide hydrochloride
GAPDH	Glyceraldehyde-3-phosphate Dehydrogenase
ANOVA	ANalysis Of VAriance
SD	Standard deviation
