# Supplementary figures and images for: Placental Barrier Breakdown Induced by Trypanosoma cruzi-Derived Exovesicles: A Role for MMP-2 and MMP-9 in Congenital Chagas Disease
Source: Int J Mol Sci. 2025 Dec 17;26(24):12131. doi: 10.3390/ijms262412131 (PMC12733037; doi:10.3390/ijms262412131)

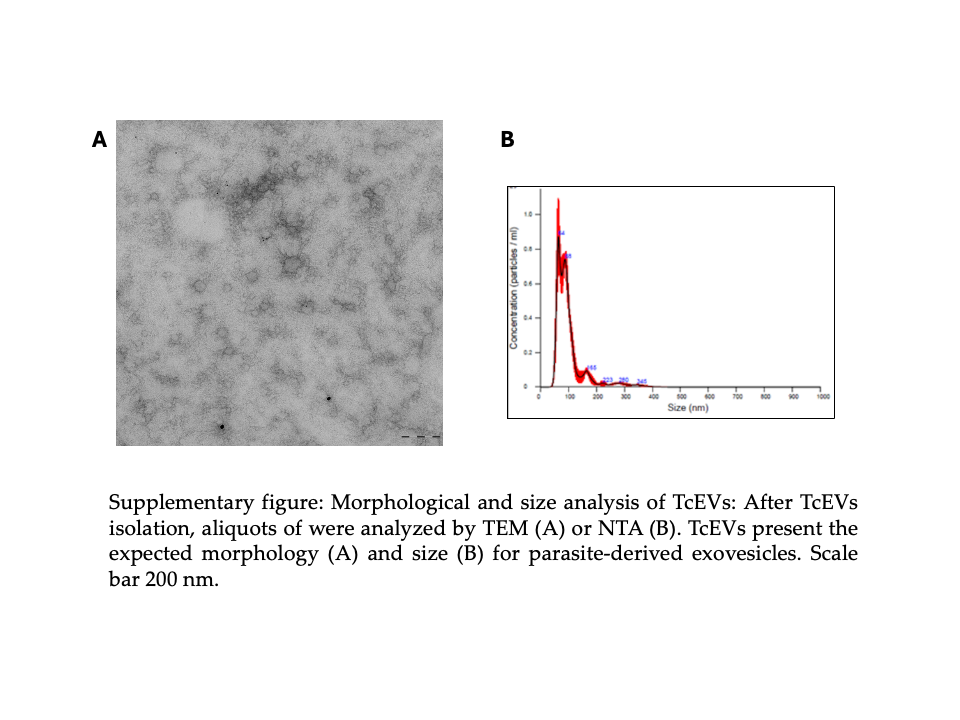

Supplement: Supplementary file 1 [file ijms-26-12131-s001.zip › ijms-3976927-supplementary.png]
